# Combined MitraClip and Left Atrial Appendage Occlusion: Is It Still a Utopia?

**DOI:** 10.3389/fcvm.2022.940560

**Published:** 2022-07-12

**Authors:** Martina Belli, Federico Zanin, Massimiliano Macrini, Lucy Barone, Massimo Marchei, Saverio Muscoli, Francesca Romana Prandi, Domenico Sergi, Marco Di Luozzo, Francesco Romeo, Francesco Barillà

**Affiliations:** ^1^Department of Systems Medicine, University Tor Vergata, Rome, Italy; ^2^Division of Cardiology, University Hospital Tor Vergata, Rome, Italy; ^3^Department of Departmental Faculty of Medicine, Unicamillus-Saint Camillus International, University of Health and Medical Sciences, Rome, Italy

**Keywords:** MitraClip, Left Atrial Appendage Occlusion, percutaneous transcatheter mitral valve repair, atrial fibrillation, combined percutaneous procedures

## Abstract

Atrial fibrillation (AF) is the most common cardiac arrhythmia, affecting 32 million individuals worldwide, particularly the elderly. It is the main cause of ischemic strokes. Oral anticoagulation (OAC) is the gold standard strategy for stroke prevention. Still, there is a not negligible share of patients who have contraindications to this therapy, more frequently due to an increased risk of bleeding. AF is often associated with moderate-severe mitral regurgitation (MR), the second most frequent valvular disease in elderly patients. Data from the literature reported that more than half of patients with severe mitral regurgitation are not suitable candidates for cardiac surgery. Given the progressive aging of the population and the simultaneous increase in the number of patients with comorbidities, the advent of new therapeutic strategies, such as the combined approach of Left Atrial Appendage Occlusion (LAAO) and MitraClip procedure, is acquiring great interest. At present, the category of patients who may benefit from combined percutaneous therapies and the long-term risks and benefits might not have been identified. Despite the efforts of researchers, the correct selection of patients is a very important clinical need that has not yet been met to avoid committing human and financial resources to interventions that may be unnecessary. It is conceivable that the most modern and recent innovations in cardiovascular imaging, particularly three-dimensional echocardiography and new methods of volume imaging, could improve our ability to select patients appropriately. Since data in the literature are scarce, future studies will be needed to evaluate the efficacy and safety of combined MitraClip and LAA occlusion.

## Introduction

Population aging brings to the attention of cardiologists more and more complex patients with multiple comorbidities; therefore, the need for combined percutaneous procedures is affirming in the field of interventional cardiology. Atrial fibrillation (AF) is the most common cardiac arrhythmia, affecting 32 million individuals worldwide, and has a high impact on the costs of the health system (1). Its incidence increases with age, reaching about 6% in people over 60 (2). It is characterized by grossly disorganized atrial electrical activity and an increased thromboembolic risk. It is responsible for 15–20% of cerebrovascular accidents of ischaemic origin. In non-valve atrial fibrillation (NVAF), 90% of blood clots form at the level of the left appendage, a small, ear-shaped sac in the muscle wall of the left atrium (3, 4). Anticoagulant therapy is the gold standard in reducing the risk of stroke, but about 1 in 10 patients do not receive anticoagulant therapy due to contraindications: previous bleeding, anemia, chronic renal failure, and liver cirrhosis (5). The current guidelines recommend anticoagulant therapy in patients with CHA2DS2-VASc score ≥2. Direct-Acting Oral Anticoagulants (DOACs) are recommended as first-line therapy in patients with NVAF, while Vitamin K Antagonist (VKAs) are used in AF patients with mechanical valve prosthesis or moderate-to-severe mitral stenosis. Lifelong dependence on anticoagulation in patients with AF is inevitably associated with an increased risk of bleeding complications as well as cardioembolic events in the event of inadequate therapy and, ultimately, significant lifestyle changes (e.g., consulting with the doctor before taking medications, certain vegetables, or supplements, regular blood tests and avoiding major dietary changes when taking VKAs) (6). Consequently, real-world data has demonstrated that the adherence to the therapy is poor, and up to 25–30% of patients stop OAC on long-term follow-up (7). AF in about 30% of cases is associated with mitral valve disease, more frequently with moderate-severe mitral regurgitation (MR) (8). MR is, after degenerative aortic stenosis, the second most frequent valvular disease in elderly patients, which, if not properly treated, has a very high mortality. More than half of patients with severe mitral regurgitation are not suitable candidates for cardiac surgery. Percutaneous transcatheter closure of the LAA and percutaneous transcatheter mitral valve repair with the MitraClip system are new therapeutic strategies in patients at high risk of hemorrhagic and cardioembolic events. The percutaneous repair procedure with the “edge to edge” technique using MitraClip is a procedure based on the same principle of surgical correction proposed by Alfieri in the early 90s: a double orifice valve is created through a permanent suture between the free margin of the two mitral flaps (9). Compared to conventional surgery, using a transcatheter antegrade approach, the MitraClip System (Abbott Vascular, Abbott Park, IL, USA) is less invasive. Percutaneous occlusion of LAA consists of the positioning of a device in the site where thrombi are most frequently allocated. Since 1949, heart surgeons have performed the combination of mitral valve repair and LAA exclusion in patients with MR and AF; the LAA occlusion is recommended in patients with AF who undergo heart surgery (10). These important clinical findings have prompted interventional cardiologists to start this combined approach percutaneously. The last ESC guidelines recommended the MitraClip procedure for symptomatic patients with severe primary or secondary MR, who are judged inoperable or at high risk for surgical repair and LAA closure, for stroke prevention, in patients with AF and contraindications for long-term anticoagulant treatment. Therefore, the purpose of this review is to evaluate the clinical aspects and advantages that can derive from performing the two procedures in the same session in selected groups of patients.

## MitraClip

The MitraClip (Abbott Vascular Santa Clara, CA, USA) is a percutaneous mitral regurgitation repair procedure that consists of the insertion of one or more clips between the anterior and posterior mitral leaflets with the formation of a double orifice valve and subsequent reduction of the degree of regurgitation. The data of the EVEREST trials (11, 12). and results of registries (13) demonstrated that the MitraClip procedure was feasible and safer than surgical mitral-valve repair but was not as effective in reducing the severity of mitral regurgitation. More recently two trials, MITRA-FR (Percutaneous Repair with the MitraClip Device for Severe Functional/Secondary Mitral Regurgitation) and COAPT (Cardiovascular Outcomes Assessment of the MitraClip Percutaneous Therapy for Heart Failure Patients with Functional Mitral Regurgitation) assessed the efficacy and safety of MitraClip in patients with systolic heart failure and severe secondary MR (14, 15). A priori, these two trials targeted the different patient populations with the same disease using the same device but the results of these trials were diametrically opposed: MITRA-FR being neutral and COAPT being highly positive with respect to the efficacy of the MitraClip procedure. MITRA-FR and COAPT targeted the same disease entity with the same device, the MitraClip. However, COAPT enrolled a subset of patients who had more severe MR and less advanced LV disease (dilation/dysfunction) compared to patients with MITRA-FR. These differences may explain the different outcomes observed in COAPT vs. MITRA-FR. Indeed, patients with too severe LV (Left ventricle), or right ventricle, dilation/dysfunction (i.e., too extensive LV myocardial damage) may not benefit from the MitraClip procedure. In view of the results of the studies MITRA-FR and COAPT, it, therefore, seems reasonable to conclude that the MitraClip procedure reduces hospitalization of patients with heart failure and mortality in patients who fulfill the following criteria: (1) ≥ moderate-to-severe (3+) secondary MR, defined as EROA ≥30 mm^2^ and/or regurgitant volume >45 ml; (2) LVEF between 20 and 50% and LV end-systolic diameter <70 mm) despite optimal (maximally tolerated) guideline-directed medical therapy (GDMT) with cardiac resynchronization and coronary revascularization if appropriate. Furthermore, the goal of the procedure should be to obtain an acute reduction of the MR severity to ≤ mild (1+) and the implantation of additional clips should be considered to achieve this goal (16). This procedure is currently indicated in patients affected by severe MR and prohibitive surgical risk, namely patients deemed not good surgical candidates for MitraClip after the discussion about the potential its feasibility (17). So the 2021 European Society of Cardiology Guidelines for the management of valvular heart disease recommended transcatheter edge-to-edge repair (TEER) with MitraClip, for patients with symptomatic, severe primary mitral regurgitation, that fulfill the echocardiographic criteria of eligibility and judged inoperable or at high surgical risk by the Heart Team (class of recommendation IIb, level of evidence B). For patients with severe secondary mitral regurgitation, the ESC Guidelines recommended MitraClip procedure for selected symptomatic patients, not eligible for surgery and fulfilling criteria suggesting an increased chance of responding to the treatment (class of recommendation IIa, level of evidence B). At the same time, in high-risk symptomatic patients not eligible for surgery and not fulfilling the criteria suggesting an increased chance of responding to TEER, the Heart Team may consider in selected cases a MitraClip procedure (or other transcatheter valve therapy if applicable), after careful evaluation for ventricular assist device or heart transplant (18). Anatomical evaluation of the mitral valve, particularly in the degenerative form, is of fundamental importance for the feasibility of the MitraClip. The main contraindications are extensive calcifications on free margins of leaflets, area <3 cm^2^, mean gradient >5 mmHg, perforation of leaflets, active endocarditis, rheumatic mitral valve disease.

## Patient Selection and Imaging Guided MitraClip Implantation

**Figure 1:**
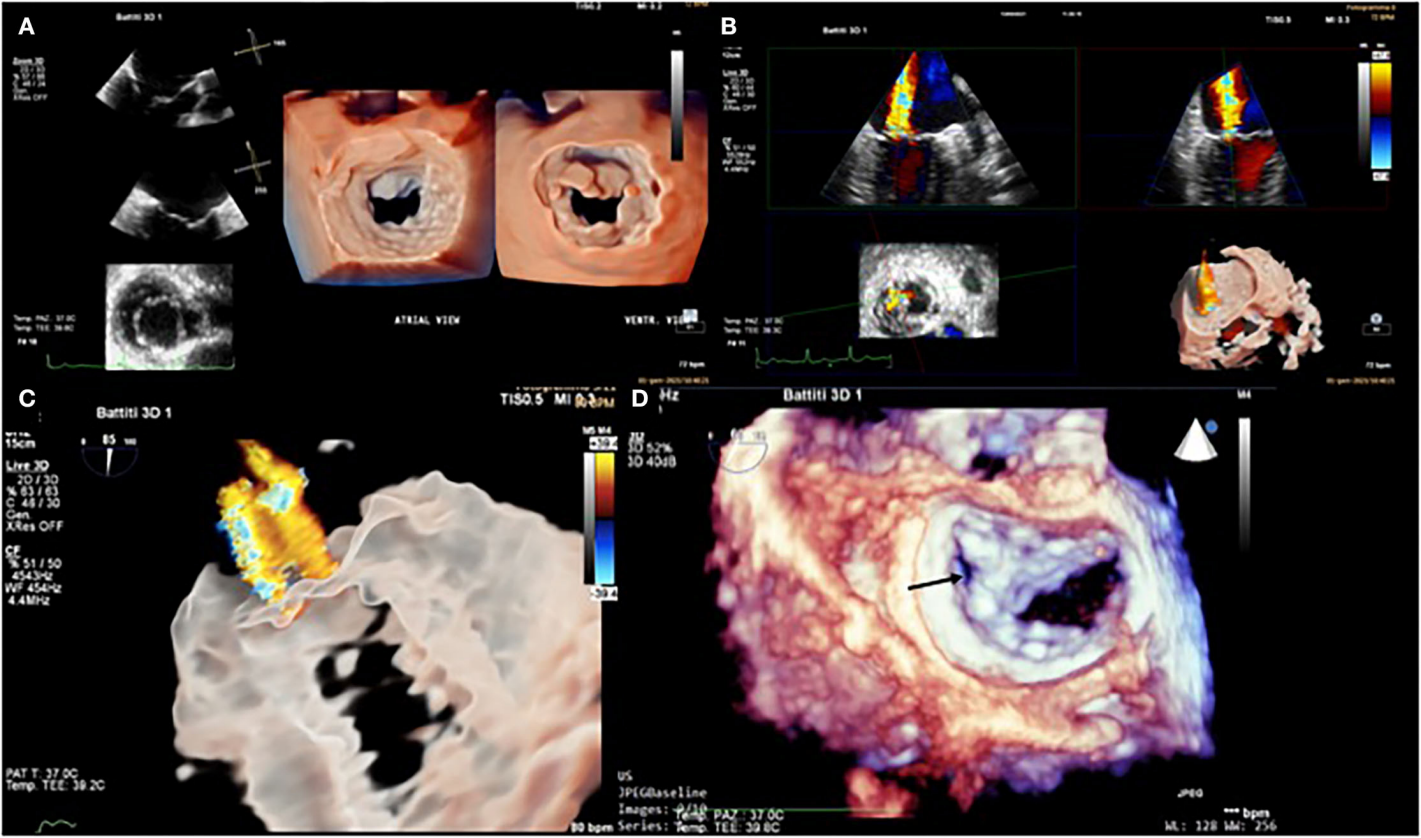
Transesophageal echocardiogram. 3D mitral valve reconstruction with atrial view (or “surgeon's view”) and ventricular view sergi **(A)**. Severe mitral regurgitation (MR) was documented with 3D color Doppler acquisition **(B)**. 3D Glass image of MR **(C)**. 3D mitral valve reconstruction (atrial view) after Mitra-Clip system placement in the lateral paracommissural region (black arrow) **(D)**.

The patient selection and pre-procedural echocardiographic evaluation, in particular, to diagnose the pathoanatomic mechanism, the severity of the mitral regurgitation, the right and left ventricular size and function, the left atrial size, pulmonary hypertension, and severity of tricuspid regurgitation are crucial to identify ideal candidates for MitraClip. We describe the procedure's steps in detail, stressing the importance of collaboration between the echocardiographer and the interventional cardiologist. Two-dimensional transesophageal echocardiography (2D TEE) is the primary imaging modality for the guidance of the procedure. Realtime three-dimensional (3D) TEE has recently been introduced as an additional imaging modality (Figure 1). In comparison with 2D TEE, 3D TEE provides additional information in some procedure steps, such as precise positioning of the clip delivery system into the left atrium, and correct alignment of the clip arms perpendicular to the coaptation line, and confirmation of the right grasping location (19). Biner et al. demonstrated that using 2D and 3D TEE in combination is associated with a remarkable 28% reduction in procedure times (20). Fluoroscopy provides additional helpful information on the positioning and distance for delivery catheter advancement and MitraClip positioning. Fluoroscopy provides good spatial and temporal resolution over a wide field of view with continuous monitoring and easy identification of devices. Still, it can only provide monoplane information, does not allow visualization of soft tissue, and is related to the use of ionizing radiation and contrast medium. The two methods are therefore complementary, and the most recent innovation, such as fluoroscopic-echocardiographic fusion imaging with the new EchoNavigator (Philips Medical System, Best, The Netherlands) and TrueFusion (Siemens Healthinners, Erlangen, Germany) systems, allows simultaneous acquisition of both fluoroscopic and echocardiographic images and co-registration, thus overcoming the limitations of the two methods. The coordinates of the two images are integrated into the same reference system, thus obtaining a hybrid image, which has the advantage of being easily interpretable by the interventional cardiologist since the echocardiographic images in which the soft tissues and the functional aspect (e.g., valve regurgitation jets) are well-visualized, are integrated in real-time in the standard fluoroscopic projections where the catheters and devices are easily identifiable (21).

## Mitraclip Procedure

The procedure can be divided into six steps:

- Trans-septal puncture (after cannulation of femoral vein);
- Introduction of the steerable guide catheter (SGC) into the left atrium;
- Advancement of the clip delivery system (CDS) into the left atrium and positioning of the MitraClip below the mitral valve leaflets;
- Crossing the valve and advancing the CDS into the left ventricle;
- Grasping the leaflets;
- Assessment of the result.

### Trans-Septal Puncture

The trans-septal approach and puncture are crucial; the latter is one of the most important aspects of the MitraClip procedure. The optimal puncture has to be superior and posterior across the interatrial septum. In degenerative MR, 4–5 cm are required from the exit point in the left atrium to the mitral annulus to allow good mobility of the system; in functional MR, the line of coaptation is usually below the plane of the mitral annulus due to extensive tethering. Therefore, the puncture site in these patients needs to be inferior and closer to the annular plane (about 3.5 cm above the annular plane). To establish the exact position of the puncture, the TEE is indispensable. The two-dimensional picture shows the so-called “tenting”, i.e., the deformation that the pressure of the catheter exerts on the fossa ovalis before the tissue is passed through. To determine the exact point where the septum is perforated, three planes are needed with the 2D TEE: the bicaval view for superior-caudal orientation, the short-axis view for anteroposterior direction, and the four-chamber view to measure the height between the exit point and the annulus plane. In contrast, the 3D TEE provides all this information in a single image: a projection of the interatrial septum similar to the fluoroscopic left anterior oblique projection, including the mitral valve, allows a realistic visualization of the “tenting” and, in the same image, easily measures the distance between the puncture site and the mitral valve annulus.

### Introduction of the Steerable Guide Catheter Into the Left Atrium

Once the septum is punctured, the guidewire is commonly positioned into the left upper pulmonary vein with care to avoid entering the left atrial appendage and the risk of perforation under fluoroscopic and TEE guidance.

### Advancement of the Clip Delivery System Into the Left Atrium and Positioning of the MitraClip Below the Mitral Valve Leaflets

When the SGC is stably positioned in the left atrium, the guide wire is replaced by CDS. Then the catheter and clip are oriented toward the mitral valve by rotating the catheter 90°. For correct orientation, 2D ETE is essential, which uses two echocardiographic views: a 2-chamber view to establish the latero-medial position of the catheter and a long axis on the left ventricular outflow tract to establish its anteroposterior position. ETE 3D, on the other hand, allows real-time monitoring of maneuvers that position the catheter perfectly perpendicular to the line of mitral valve coaptation using a single 3D en face view.

### Crossing the Valve and Advancing the CDS Into the Left Ventricle

Routinely, when the catheter is perpendicular to the valve plane, operators open the clip arms and orient them perpendicularly to the coaptation line of the leaflets. This step is critical as it will allow easier and faster grasping. In the routine practice, it is recommended to pass the opened clip into the left ventricle (22), many operators prefer to advance the clip closed, like Sherif et al. (23) at the site of the regurgitant jet under the mitral leaflets in the LVOT view under breath holding, because crossing the mitral valve with an open clip can cause it to rotate during translation from the left atrium to the left ventricle, prolonging the procedure.

### Grasping the Leaflets

When the MitraClip is in a satisfactory position, the gripper is opened so as to grasp the leaflets between the grippers and the arms. This phase of the procedure is driven by the 2D ETE: identifying valve leaflets trapped between the grippers and the arms requires a high spatial and temporal resolution that 3D ETE does not yet possess. Once captured, the 3D ETE displays the clip attached to the two leaflets from a ventricular view, from which it is possible to evaluate the new morphology of the valve with the two neo-orifices. The presence of significant regurgitation may require a second clip. If the mean gradient is >5 mmHg and valve area <2 cm^2^, this is contraindicated due to the risk of mitral stenosis.

### Assessment of the Result

Residual mitral regurgitation with color Doppler is performed by rotating the TEE probe medially (clockwise) and laterally (anticlockwise) relative to the view showing the MitraClip. The pulmonary vein flow pattern is assessed with the aim of achieving a systolic dominant pattern. Intraprocedural monitoring of left atrial pressure and the presence of V waves is also commonly performed to assess the success of reducing or eliminating significant mitral regurgitation (17). The mitral regurgitation quantification is dependent on pre and after-load of the left ventricle, general anesthesia, and whether inotropic or vasopressor drugs are used. This is especially the case for functional mitral regurgitation. In routine practice, 2D color Doppler imaging allows visual detection of residual mitral jets. The exact quantification of the grade of mitral regurgitation due to these multiple jets has yet to be validated, but their extension into the left atrium is routinely used. Then, an anatomical assessment of the valve using 3D echocardiography is performed to confirm that both leaflets are correctly and symmetrically joined by the clip or clips. If the result is satisfactory the clip is released, the delivery sheath is withdrawn back and the procedure is completed. At this point, it is essential to assess the size and the direction of shunting of the iatrogenic atrial sept defect because it appears that persistent interatrial shunting is associated with worse clinical outcomes and mortality (24).

## LAA Anatomy and Indication for Closure

**Figure 2:**
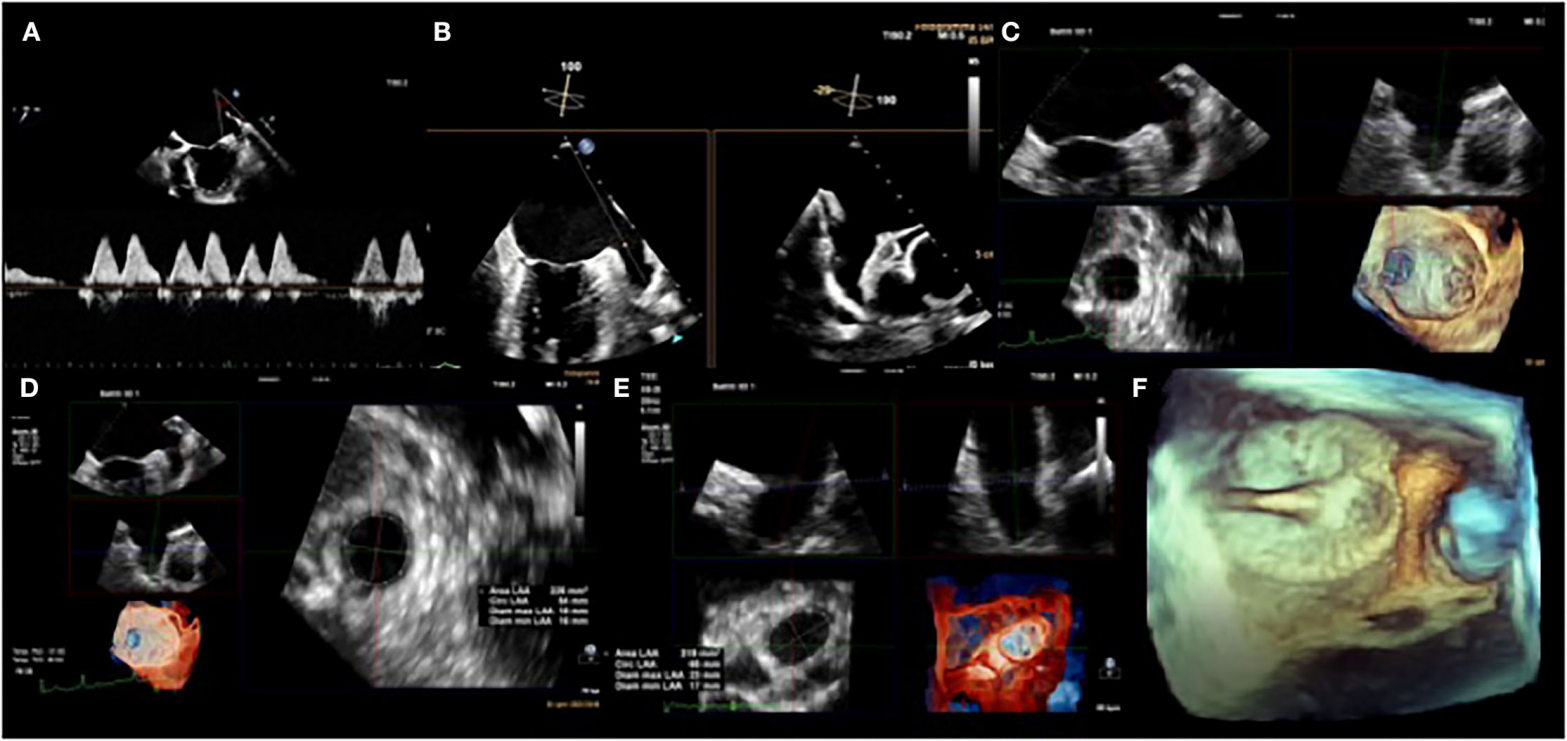
Transesophageal echocardiogram. Left atrial appendage (LAA) flow velocity pattern by pulsed-wave Doppler **(A)**. 2D TEE biplane LAA assessment. Note how the appearance of the appendage varies in the different views **(B)**. 3D TEE evaluation of the LAA landing zone **(C)**, with post-processing analysis measurements of the diameters and the area of the LAA **(D,E)**. 3D colorized depth map of LAA occluder device using peri-operative TEE **(F)**.

Left Atrial Appendage (LAA) is mainly responsible for the formation of a thrombus that can cause strokes by embolization of the cerebral circulation (3). LAA is a structure derived from the primordial left atrium (LA), which develops during the third to sixth week of fetal cardiac development from the pulmonary venous bud and it has unique physiological characteristics (25). First of all, LAA is more compliant than the left atrium and has an important role in LA decompression during overload. The occlusion of LAA determined an improvement in the LA reservoir and conduit function. Moreover, LAA has an endocrine role. It accounts for the production of atrial natriuretic peptides (ANP) that contribute to natriuresis and diuresis. The distension of LAA is directly correlated with the production of ANP rather than elevation in the LA pressure or distension of the body of LA. For this reason, LAA closure can downregulate Renin-Angiotensin-Aldosterone System and Adrenergic Input (26). There are several morphological LAA classifications. The most commonly adopted one consists of four shapes: chicken-wing (~48%; presence of a significant bend), windsock (~19%; single dominant lobe without a significant bend), cactus (~30%; dominant central lobe with multiple secondary lobes), and cauliflower (~3%; short LAA without a dominant lobe that branches into several lobes) (27). The shape of the LAA may affect stroke risk. In particular, the presence of extensive trabeculations is correlated to higher risk. Furthermore, the LAA shape can increase the technical challenge for percutaneous LAA closure. Therefore, imaging is essential to pre-plan equipment selection and implantation strategy, to guide procedural device implantation, and also for device surveillance post implantation. The transesophageal echocardiography 2D (2D TOE) is routinely used in assessing LAA morphology, and recent 3D innovations such as multiplanar reconstruction (3D TOE MPR) and 3D TOE TrueView Glass rendering (Figure 2), have increased its accuracy. However, the gold standard in defining the morphology of the LAA remains the computed tomography (CT). LAA occlusion provides an alternative to oral anticoagulation for thromboembolic risk reduction in patients with nonvalvular atrial fibrillation (28). The earliest study was made by Madden et al. in 1948, which showed that the LAA was a source of thrombus formation in patients with AF and that its removal could prevent systemic thromboembolism (29). Subsequently, surgeons started to exclude progressively, the LAA during mitral valve intervention, in patients with AF. Over the years, many devise has been developed for the closure of the LAA by percutaneous interventional techniques. The first device designed for percutaneous LAA occlusion was the PLAATO device (Appriva Medical, Sunnyvale, California) (30). The device is made by a self-expanding nitinol cage covered by a polytetrafluoroethylene membrane. Despite the promising results, this device was taken off the market in 2016 (31). Currently, many percutaneous LAA closure devices have obtained CE mark. Watchman (Boston Scientific, Marlborough, MA, USA) and the Amplatzer Cardiac Plug (ACP) (Abbott, St Paul, MN, USA) are the most commonly used devices for mechanical orifice obstruction, and the Lariat device (SentreHEART, Redwood City, CA, USA) for epicardial suture ligation. The Watchman device is approved in many countries worldwide and is the only device studied in randomized trials, as well as in multicenter prospective non-randomized studies. The PROTECT AF (Watchman Left Atrial Appendage Closure Device for Embolic Protection in Patients with atrial Fibrillation) trial, a multicenter prospective RCT, established that the percutaneous LAA closure device was non-inferior to warfarin for the combined primary efficacy endpoint of cardiovascular mortality, all-cause mortality, and systemic thromboembolism (32). The PREVAIL (Prospective Randomized Evaluation of the Watchman Left Atrial Appendage Closure Device in Patients With Atrial Fibrillation vs. Long Term Warfarin Therapy) trial, was conducted to assess the safety and efficacy of LAA occlusion raised from the PROTECT AF study. The study concluded that LAA occlusion was non-inferior to warfarin for ischemic stroke prevention (33). For ACP and its second-generation, Amulet, multiple retrospective and prospective registries have reported successful device implantation in 95–100% of patients, with a low rate of major periprocedural adverse events. A recently published, industry-initiated, large, randomized, multi-center, trial (Amulet IDE trial), evaluating the safety and effectiveness of the Amulet occluder compared with the Watchman™ device, has shown that the Amulet occluder was non-inferior to safety and effectiveness of stroke prevention compared with the Watchman device (34). In light of the results of these trials, ESC guidelines suggest LAA closure, for stroke prevention, in patients with AF and contraindications for long-term anticoagulant treatment, e.g., intracranial bleeding without a reversible cause (class of recommendation IIb, level of evidence B) (35).

## LAA Implantation Technique

The LAA closure is implanted by a percutaneous procedure and is typically performed under general anesthesia or conscious sedation. Is reasonable to perform closure under general anesthesia to ensure patient immobility and transesophageal monitoring. Intravenous or oral antibiotic prophylaxis is administered once before and also after the procedure. Right femoral access, under ultrasound or fluoroscopic guidance to avoid vascular complications, is recommended. It is preferred over left access, to give more stability to transeptal puncture. One of the most critical parts of the procedure is the transeptal puncture. It can be performed with traditional transseptal systems such as an SL-1 sheath and Brockenbrough (BRK) needle. It can be guided under fluoroscopy guidance but it is strongly recommended to perform under echographic (ICE or TEE) vision; as for the other procedures “fusion” could provide an added value: the preferred site is the postero-inferior portion of the fossa ovalis which ensures maximum co-axiality between the long axis of the auricle and the catheter. This ensures the best orientation of the catheter during device release to avoid as much as possible movement of the catheter within the LAA, a potential cause of wall damage. If a patent foramen ovale (PFO) or an atrial septal defect is present is reasonable to use that access to the left atrium. In this case, the access will be more anterior and the approach to the LAA can be more difficult. It is crucial to administer heparin (before or upon transseptal crossing for a target activated clotting time (ACT) of >250 s.

Once access to the left atrium is obtained, a wire is advanced into the left superior pulmonary vein and subsequently, a 14F access sheath is exchanged and it is advanced into the left atrium.

A pigtail catheter is advanced in the access sheath to reach LAA and perform angiography. Is important to position the pigtail catheter tip into the deepest lobe of LAA. A two orthogonal views angiography can be used to confirm the sizing of the device. It is advisable to oversize by 20% the diameter of the device to larger than the largest size to decrease the risk of leaks.

Once a device has been chosen, the pigtail catheter remains in the LAA to maintain its position and prevent the advancement of the sheath. Once the device is prepared and ready for insertion, the pigtail catheter can be removed and the delivery catheter can be inserted. The procedure is followed under fluoroscopy usually using the cranial RAO projection on which a 3D volume or 2D images TEE can be superimposed. This allows appreciation of the long axis of the LAA, the Coumadin ridge, and the left superior pulmonary vein, and the movement of the guidewire as it passes from the left superior pulmonary vein into the LAA. The delivery catheter and the access sheath will be connected and then both are pulled to unfold the device. During the deployment of the device is recommended to hold the breath or the ventilation to find the best position. Then the device is opened and the correct anchorage is verified with the “tug test”. When the device is fully unfolded four elements (PASS criteria: P = position, A = anchor, S = size, S = seal), will be checked in fluoroscopy and TEE. If all four PASS criteria are met, the device can be released by rotating the delivery cable counterclockwise. A contract angiography of the left atrium can be performed to find the peri-device leak. Then the delivery cable can be retracted into the access sheath and removed. Femoral access can be closed by compression or vascular closure devices (36).

## Combined Intervention of MitraClip and LAA Closure

The interest in combined percutaneous procedures is increasing in the field of interventional cardiology, especially in complex patients with multiple comorbidities. Percutaneous transcatheter mitral valve repair with the MitraClip system and left appendage occlusion (LAAO), are new therapeutic strategies in older patients with severe mitral insufficiency and atrial fibrillation that are at both high-risk of cardioembolic events and bleeding.

Currently, limited evidence exists regarding the combined procedure of MitraClip and LAAO and is mainly derived from single clinical cases or small sample trials (maximum 25 patients enrolled) (37, 38), showing the technical feasibility and safety of the combined procedure.

The two combined procedures could have important advantages: first of all, a single trans-septal approach can reduce the risk of complications compared to double puncture, although at different sites. Furthermore, the use of large sheaths in two different positions may increase the risk of significant residual septal shunting (39). In addition, single venous access would be required with less risk of bleeding; the procedure and fluoroscopy times would be reduced as the initial steps are the same and this could also reduce the length of hospital stay. Certainly, there are some doubts about the position of the trans-septal puncture, since for the MitraClip it is indicated in the postero-superior position, but for LAAO it is indicated in the postero-inferior position of the fossa ovalis, so there is a risk of not having a correct alignment for the second procedure. Previous reports have demonstrated the feasibility and safety of LAAO in combination with MitraClip (40). The MitraClip procedure is generally performed before LAAO because it is more technically demanding. In addition, the device's presence in the LAA could lead to technical difficulties in the introduction of the MitraClip, especially in the phase of steering and rotation of the device in the left atrium. In the LAAO procedure, dedicated deflectable catheters can help overcome the lack of alignment and allow correct positioning of the prosthesis. However, if the coaxial LAA approach is impossible, a second transeptal should be performed to achieve an optimal LAAO. Another tricky aspect is femoral venous access, as a 24F delivery sheath is required for the MitraClip, and a 14F for LAAO, so if the MitraClip is done first, effective hemostasis must be ensured e.g., with Proglide systems to close the orifice around the sheath and reduce the risk of bleeding. So, in what order should the two procedures be carried out? Francisco et al. (39) suggested performing the LAAO first rather than the MitraClip which would require an exchange for a shorter sheath compatible with the 14F Watchman delivery sheath, to avoid massive bleeding at the access site and they found that the presence of the device in left appendage served as a useful anatomical reference during manipulation of the MitraClip delivery system. On the contrary, D'Amico et al. (41), performed the MitraClip first, favoring transseptal puncture, and then to ensure effective hemostasis by switching from a larger to a smaller caliber guide catheter, completed the intervention with LAAO previous an eight-lumen suture. Therefore, from a clinical standpoint, patients referred for MitraClip implantation frequently present a profile suitable for LAA occlusion and conversely. The combined approach has advantages: a single procedure involves a single transseptal puncture and single vascular access reducing the risk of complications, finally, overall fluoroscopy time may be reduced compared to two individual procedures. On the other hand, there are disadvantages: the high trans-septal puncture for the MitraClip is less well-suited for LAA occlusion and the overall procedure time may be prolonged, with an added risk of volume overload or hemodynamic instability, especially considering the severely depressed systolic function of many of these patients. A combined MitraClip and LAAO procedure appear to be feasible and safe, with a favorable medium-term outcome of thromboembolism, bleeding, and heart failure hospitalization. The positioning of embolic brain protection systems *via* trans-radial would also allow reducing complications associated with prolongation of the combined procedure (42).

## Clinical Implications

The combined approach of MitraClip and LAAO is a great stimulating innovation in interventional cardiology, as through a minimally invasive approach and with relatively acceptable risk, it allows to treat elderly patients with both diseases in which the intervention of reparative surgery is contraindicated and in which due to high bleeding risk, there's a contraindication for systemic anticoagulation. In most cases, these are complex patients with chronic diseases, with important comorbidities and therefore in polypharmacy, in whom ischaemic and hemorrhagic risk assessment is essential. However, patient selection is of crucial importance for the success of the two combined interventional procedures.

## Conclusion

Percutaneous MitraClip intervention and LAA closure are generally recommended procedures for patients with severe mitral insufficiency and AF, who are at high risk of both surgical valve repair and bleeding during anticoagulation treatment. Certainly, it is a very interesting area that could offer opportunities to treat fragile patients, guaranteeing an improvement in symptoms, functional capacity, and quality of life. Thanks to the latest imaging innovations and more sophisticated interventional techniques, combined treatments in the same session would not seem utopian and could be the best strategy for selected high-risk patients. However, there are still insufficient data in the literature; therefore, further studies and maybe RCT will be needed to evaluate the safety and the long-term efficacy of the MitraClip and LAAO combined approach.

## Author Contributions

MB and FB contributed to manuscript conceiving and revision. FZ, MMac, LB, MMar, SM, MD, DS, and FP contributed to data collection and literature review. All authors contributed to the article and approved the submitted version.

## Conflict of Interest

The authors declare that the research was conducted in the absence of any commercial or financial relationships that could be construed as a potential conflict of interest.

## Publisher's Note

All claims expressed in this article are solely those of the authors and do not necessarily represent those of their affiliated organizations, or those of the publisher, the editors and the reviewers. Any product that may be evaluated in this article, or claim that may be made by its manufacturer, is not guaranteed or endorsed by the publisher.
